# Effects of ocean eddies on the tropical storm Roanu intensity in the Bay of Bengal

**DOI:** 10.1371/journal.pone.0247521

**Published:** 2021-03-05

**Authors:** Yujun Liu, Haibin LÜ, Honghua Zhang, Yusheng Cui, Xueting Xing

**Affiliations:** 1 Jiangsu Key Laboratory of Marine Bioresources and Environment / Jiangsu Key Laboratory of Marine Biotechnology, Jiangsu Ocean University, Lianyungang, Jiangsu Province, China; 2 Co-Innovation Center of Jiangsu Marine Bio-industry Technology, Jiangsu Ocean University, Lianyungang, Jiangsu Province, China; 3 Jiangsu Institute of Marine Resources Development, Lianyungang, China; 4 Lianyungang Meteorological Bureau, Lianyungang, Jiangsu Province, China; Universidade de Aveiro, PORTUGAL

## Abstract

A tropical storm (TS) Roanu occurred in northern Sri Lanka in 2016, which transported northwards along the west coast of the Bay of Bengal (BoB). During the development of the TS, ocean eddies on its track had an important effect on the intensity of Roanu. The dynamic mechanism was investigated with multisource reanalysis and Argo float data in this study. The results show that ocean eddies were the main reason why Roanu first enhanced, weakened, and then enhanced again. Warm eddy W1 supports the initial development of the TS, cold eddy C1 weakens Roanu, and warm eddy W2 continues to support Roanu. On May 19, 2016, the maximum average latent heat flux over W1 was 260.85 w/m^2^, while that of C1 was only 200.71 w/m^2^. After the passage of Roanu, the tropical cyclone heat potential (TCHP) of eddies significantly decreased. The TCHP of W1, W2, C1 and C2 decreased by 20.95 kJ/cm^2^, 11.07 kJ/cm^2^, 29.82 kJ/cm^2^, 9.31 kJ/cm^2^, respectively. The mixed layer of warm eddies deepened much more than that of cold eddies, supporting Roanu development. In addition, changes in potential vorticity (PV) values caused by the disturbance of eddies may also reflect changes in the TS intensity. This study offers new insights on the influence of ocean eddies in regulating the development of tropical cyclone (TC) in the BoB.

## Introduction

**Fig 1:**
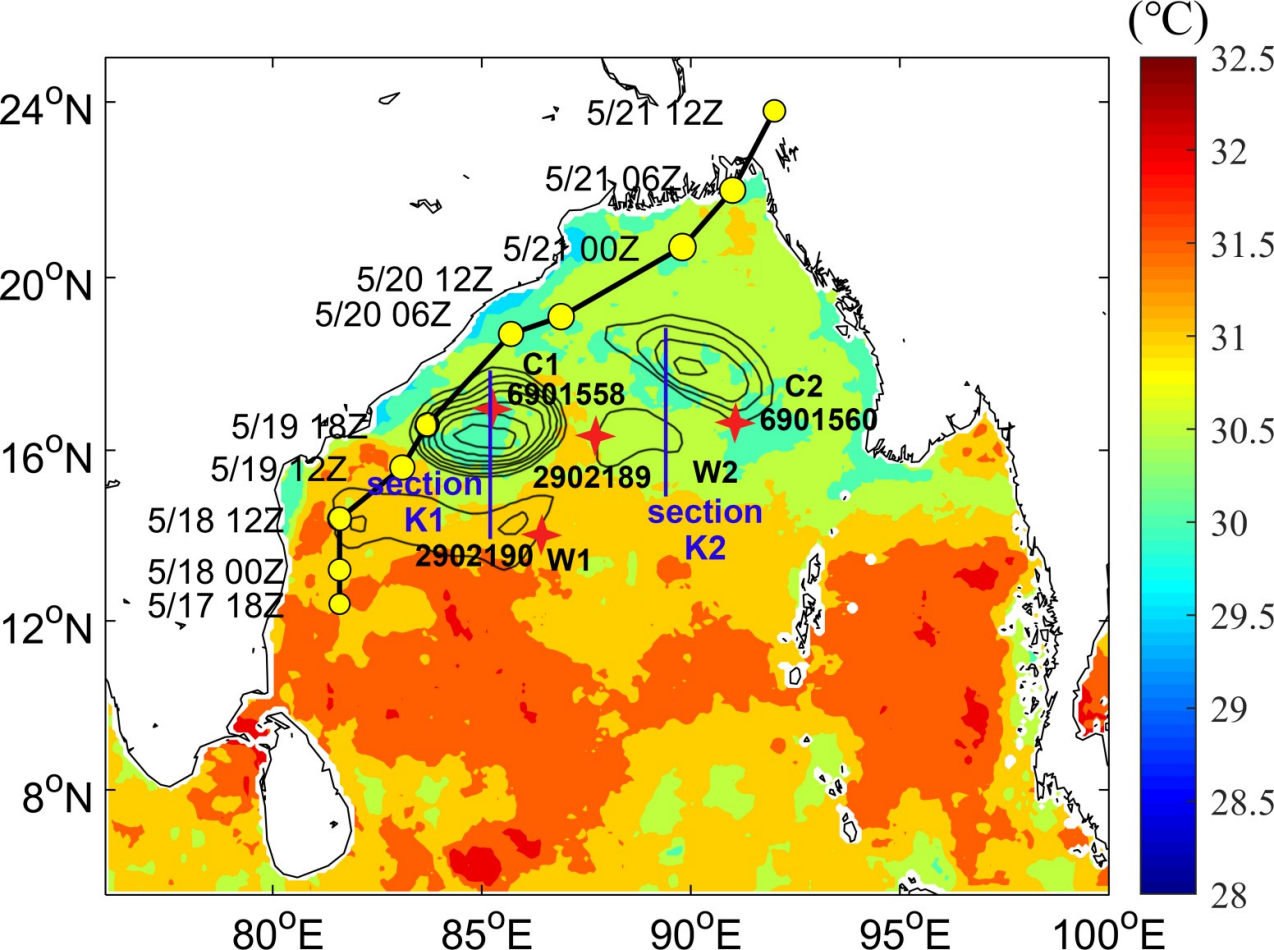
The average SST in the BoB one week before TS formation. TS track, ocean eddies and Argo floats are the positions during the passage of TS. The black line and yellow circles show major changes in the track and intensity of the TS, respectively. Black circles represent warm eddies W1, W2 and cold eddies C1, C2. Four in situ Argo floats (red asterisks) correspond to the four eddies, and their platform numbers are 2902190, 2902189, 6901558 and 6901560, respectively. The blue lines are section K1 (position: 85°E, 14–18°N) and section K2 (position: 89°E, 15–19°N), section K1 through eddies W1, C1 and section K2 through eddies W2, C2.

A tropical cyclone (TC) is a strong cyclonic vortex with a warm central structure commonly found over tropical and subtropical oceans. As a TC organizes, it will gradually develop into a tropical storm (TS), which is generally accompanied by strong winds, heavy rain, and storm surges, causing severe disasters in affected areas [1]. Although less than 7% of global TCs occur in the Bay of Bengal (BoB), 80% of the world’s deadliest cyclones form in this bay [2]. TC activity in the BoB is highly seasonal, and TC is strongest after the monsoon season (October to December) as well as being stronger before the monsoon season (March to May) [3, 4]. The formation mechanisms of TCs are quite complex, and Gray [5] proposed that several conditions must be present for one to form: the existence of a low-level vortex, a large enough Coriolis force, less vertical wind shear between the upper and lower troposphere, SST in excess of 26°C, an unstable atmosphere, and higher humidity in the middle troposphere. TC intensity is affected by several complex physical processes, including TC internal dynamics [6–8], environmental flow fields [9–11], and air-sea interactions[12–14]. Air-sea interactions play an important role in the development of a TC. A TC continuously absorbs energy and water vapor from the ocean through sensible and latent heat exchange at the air-sea interface [15]. Merrill [16] proposed that the upper limit of TC intensity may be affected by sea surface temperature (SST), and Emanuel [17] and Holland [18] developed the maximum potential intensity (MPI) theory for a TC. Jacob and Shay [19] and Shay and Uhlhorn [20] proposed that dynamic and thermodynamic responses at the mixing depth affect the structure and intensity of a TC. Some studies have shown that warm eddies can promote the intensification of a TC [21–24]. Ali et al. [25] found that the intensification of two TCs in the BoB to be related to the sea level anomaly (SLA). However, the influence of several consecutive ocean eddies on TC intensity has been less explored. The BoB is a semi-closed basin with a unique marine ecological environment [26]. Several rivers inject a large amount of fresh water into the BoB, and both fresh water and monsoons have strong impacts on ocean stratification in the area, forming a shallow mixed layer and thicker thermocline [27–29]. SST was generally high in May [30, 31], forming a warm pool (Fig 1) and creating favorable conditions for the generation of TCs. Fig 1 shows the presence of obvious ocean eddies in the BoB at this time.

A TS formed in northern Sri Lanka on May 17, 2016 and then developed gradually along the Indian Peninsula and was officially named Roanu at 12Z on May 19. Interestingly, Roanu’s intensity did not continue to enhance thereafter, weakening at 18Z on May 19 and then resuming enhancement at 06Z on May 20 and rapidly weakening at its landfall in Bangladesh on May 21 (Table 1). Therefore, this paper mainly focuses on explaining Roanu’s strengthening, weakening, and final enhancement. In section 2, a brief description of the data and methods used is given, which is followed by a description of the basic air-sea environment of the TS in section 3. The unique phenomenon studied is discussed in section 4. Concluding remarks are made in section 5.

**Table 1:** Main moving tracks of tropical storm Roanu.

Date	Lon (°N)	Lat (°E)	MWS^a^ (knots)	MLSP^b^ (mb)
5/17/2016 18Z developed into a tropical storm	81.6	12.4	35	996
5/18/2016 0Z	81.6	13.2	40	993
5/18/2016 12Z	81.6	14.4	45	989
5/19/2016 12Z developed into Roanu	83.1	15.6	50	985
5/19/2016 18Z	83.7	16.6	45	989
5/20/2016 06Z	85.7	18.7	50	985
5/20/2016 12Z	86.9	19.1	55	982
5/21/2016 00Z	89.8	20.7	60	978
5/21/2016 06Z	91	22	55	985
5/21/2016 12Z	92	23.8	40	993

Note: Times are given in UTC time (denoted as Z).

MWS^a^: Maximum wind speed. MLSP^b^: Minimum sea level pressure.

## Data and methods

### Data

Atmospheric reanalysis data on geopotential heights, wind components and PV used in this paper were mainly obtained from the ER5 data set of the European Centre for Medium-Range Weather Forecasts (ECMWF). ER5 is the latest hourly generation data product (https://www.ecmwf.int/) provided by the ECMWF with a horizontal resolution of 0.25°× 0.25°. SST was optimally interpolated with a daily measurement product provided by Remote Sensing Systems (http://www.remss.com/), the product combines microwave and infrared data, and its spatial resolution is set to 0.25°. Flow field and Mixed Layer Depth (MLD) data of a spatial resolution of 0.083°× 0.083° were obtained from the GLORYS12V1 product provided by the Copernicus Marine Environment Monitoring Service (CMEMS) (https://marine.copernicus.eu/). SLA data was obtained from AVISO (https://www.aviso.altimetry.fr/data.html) with a spatial resolution of 0.25° [32, 33] and it was derived from Altika Drifting Phase, OSTM/Jason-2, Jason-3, and Sentinel-3A altimetry measurements. Latent heat flux values were obtained from the newly released Ifremer flux v4 data set in 2018 (https://wwz.ifremer.fr/oceanheatflux/), which generates daily latent heat flux data of a spatial resolution of 0.25° based on the results of the European Space Agency’s (ESA’s) Ocean Heat Flux (OHF) project [34].

In situ data were obtained from the Argo program and specifically from Argo platforms 2902190, 2902189, 6901558, and 6901560 (http://www.argo.ucsd.edu) [35, 36]. The four floats were located at ocean eddies in the track of the Roanu tropical storm, providing temperature and salinity data for before and after the storm’s passage on May 16 and May 21, 2016, respectively.

Best track data for the tropical storm (Table 1) were obtained from the Joint Typhoon Warning Center (JTWC) (https://www.metoc.navy.mil/jtwc/jtwc.html?north-indian-ocean).

### Methods

The Ekman pumping velocity (EPV) induced by the wind field is an important index for measuring the vertical movement of the upper ocean [37]. The EPV can be calculated from the wind vector using Eq (1) and Eq (2):

$$EPV=\mathrm{curl}\left(\frac{△\times \tau }{\rho f}\right)$$ (1)

$$\tau =\rho_{a}C_{d}u^{2}$$ (2)

Where △×*τ* is the curl of surface wind stress, *ρ* is seawater density, and *f* = 2*ω sin θ* is the Coriolis parameter for latitude *θ* and Earth rotation rate *ω*. Wind stress △×*τ* is calculated using Eq (2), *ρ_a_* is air density, *C_d_* is the speed-dependent drag coefficient for neutrally stable conditions and *u* is wind velocity at 10 meters above sea level.

Compared to SST, the 26°C isotherm depth (D26) can better reflect the ocean heat content (OHC) of the upper ocean [38]. Leipper and Volgenau [39] used OHC to describe the heat loss of the upper ocean, which also known as the TCHP.

$$TCHP=\rho C_{p}\int_{0}^{D_{26}}(T-26)dZ$$ (3)

Where *ρ* is the density of the seawater, *C_p_* is the specific heat capacity of seawater under pressure, *T* is the temperature at *dZ*, and *D*_26_ is the 26°C isotherm depth.

The Brunt-Vaisala frequency represents the stability of sea water to vertical displacements such as those caused by convection [40] and is estimated by Eq (4) and Eq (5).

$$N=\sqrt{gE}$$ (4)

$$E=\frac{1}{\rho }\left(\frac{d\rho }{dz}\right)$$ (5)

Where *N* is the Brunt-Vaisala frequency, and *g* is gravitational acceleration. *E* is seawater stability calculated from seawater density *ρ* and depth z.

## Results

### Atmospheric environment

**Fig 2:**
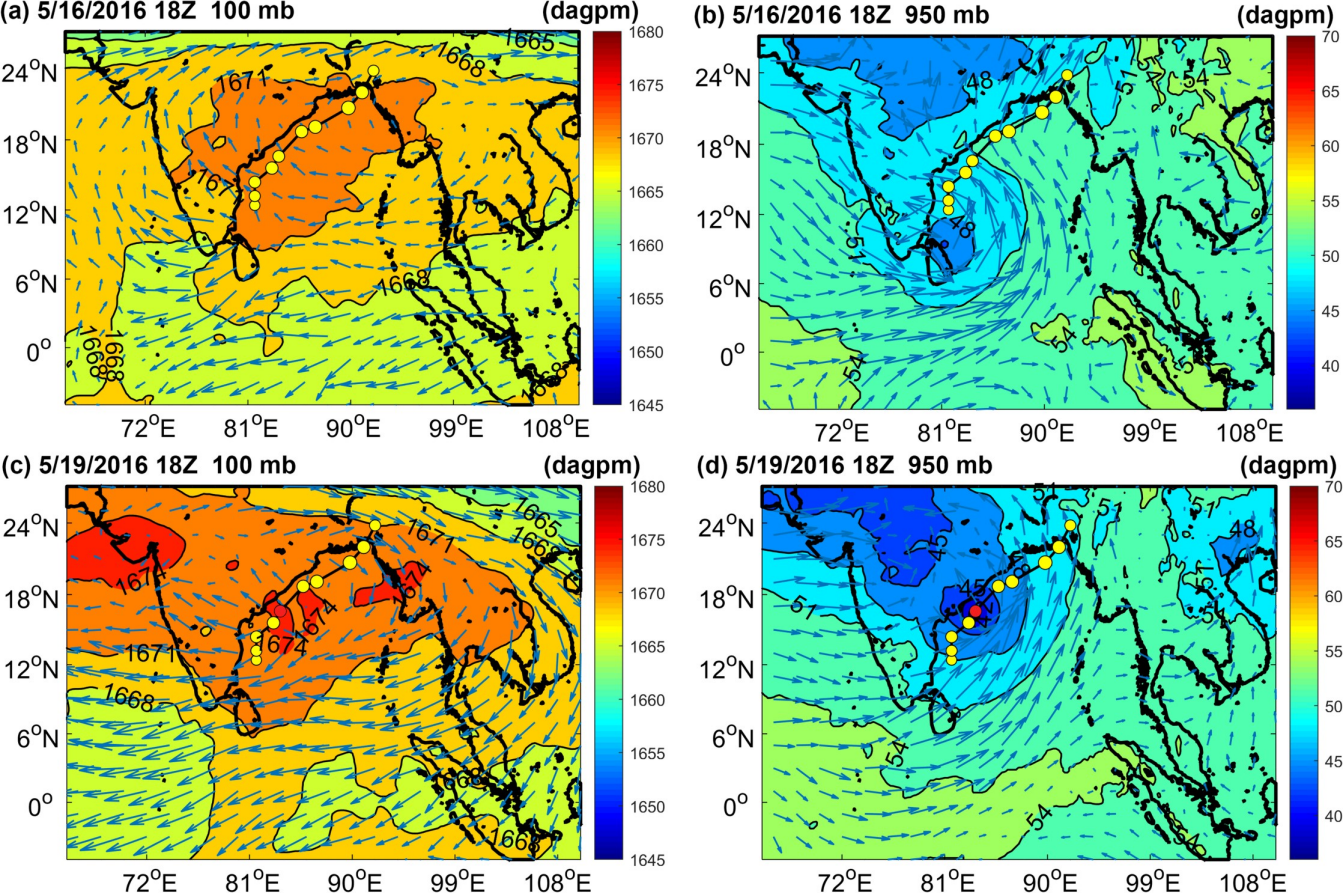
The geopotential height and wind field were distributed at 18Z on May 16 and May 19, 2016. Color bars denote geopotential heights (unit: dagpm, 1 dagpm = 10 gpm) and red dots indicate the positioning of the TS at 18Z on May 19, 2016. (A) and (C) show atmospheric pressure at 100 mb, (B) and (D) show atmospheric pressure at 950 mb.

Fig 2 shows the distribution of the geopotential height field and wind field of TS Roanu at 18Z on May 16 and May 19, 2016. TC was a low-pressure vortex, Roanu could be observed obviously in wind pattern at 950 mb (Fig 2B and 2D). The effect of upper-air system on TC could be observed at 100 mb. As seen from Fig 2A and 2C, upper levels were controlled by high pressure because of the system creating divergence aloft in the BoB sea area, and BoB was surrounded by the 1671 dagpm contour line at 100 mb (Fig 2A). The main high-pressure section develops anticyclonic circulation, and its center was greater than 1674 dagpm, which was located on Roanu’s development track at 18Z on May 19 (Fig 2C). The upper-air system provided favorable upper divergence conditions for tropical storm enhancement. A similar phenomenon also appeared in Atlantic tropical cyclone genesis events [41], Fig 2B and 2D show the geopotential height field and wind field distribution of the TS at 950 mb. BoB was mainly controlled by low pressure levels at 950 mb, forming an obvious trough was surrounded by the 51 dagpm isoline (Fig 2B). The low-pressure center was also located on Roanu’s development track, and it was less than 42 dagpm at 18Z on May 19 (Fig 2D). The wind field showed obvious signs of cross-equatorial flow (CEF) near the equator [42] and Somali Jet, facilitating the development of the tropical storm.

In general, atmospheric upper divergence, low levels of convergence and the wind field led to a favorable environment for Roanu’s strengthening, but the TS greatly weakened at 18Z on May 19, 2016.

### Marine environment

**Fig 3:**
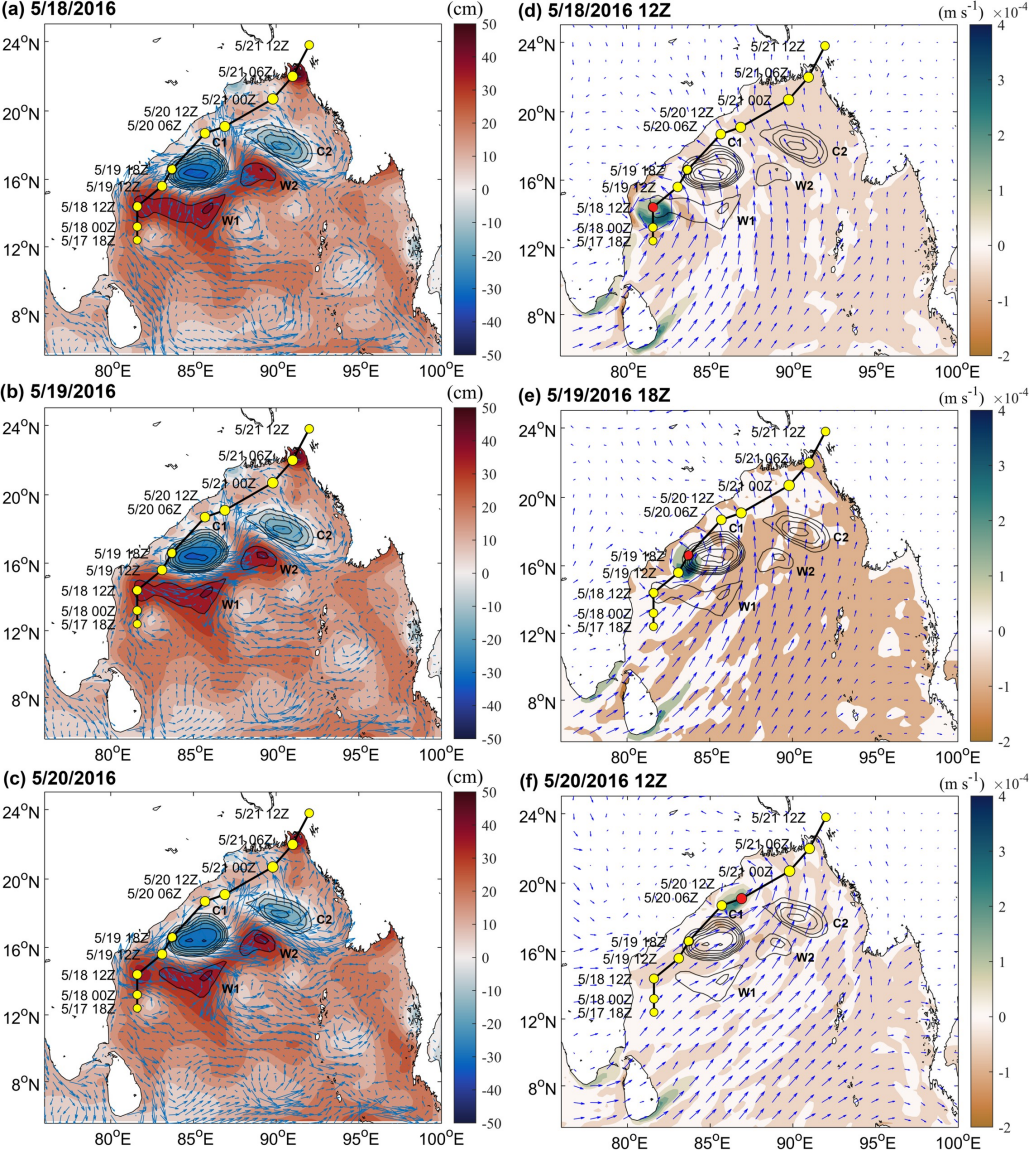
Changes in marine environmental fields during the development of the TS. (A)-(C) show the change in the SLA (color bar) and flow field (arrow) on May 18, May 19 and May 20, 2016, respectively; (D)-(F) show the change in EPV (color bar) and wind fields (arrow) at 12Z on May 18, at 18Z on May 19 and at 12Z on May 20, respectively. The positive (negative) values indicate upwelling (downwelling).

The maximum SLA value of W2 increased from 40.26 cm on May 18 to 42.02 cm on May 20, 2016 (Fig 3A–3C), and its amplitude increased from 4.05 cm to 7.7 cm from May 16 to May 21 (Table 2). The amplitude of W1 declined from 8.45 cm to 4.69 cm, and that of C1 and C2 decreased by 5 cm and 2.38 cm from May 16 to May 21, respectively (Table 2). The SLA extremum of C1 decreased from -32.04 cm on May 18 to -30.52 cm on May 20, 2016 (Fig 3A–3C). Warm eddy W2 was enhanced due to the northeast movement of the warm eddy W1 core. The enhancement of W2 better supported the re-strengthening of TS. Fig 3D–3F shows changes in Ekman pumping occurring during the development of the TS. The intensity of upwelling generated during the TS’s formation was generally low even when the maximum EPV reached 4.44 × 10^−4^ m/s at 18Z on May 19. The influence range of Ekman upwelling was limited and Ekman downwelling appeared locally in the ocean eddies. Obvious downwelling in eddies during the TS may have reduced cold-water upwelling and heat loss in ocean eddies, maintaining heat supplies to the TS.

**Table 2:** Amplitude changes of ocean eddies during the TS (unit: Cm).

Date	C1	C2	W1	W2
05/16	-27.58	-15.12	8.45	4.05
05/17	-25.88	-13.96	7.58	5.07
05/18	-24.85	-14.4	6.55	6.1
05/19	-26.89	-14.18	6.13	6.31
05/20	-25.45	-13.76	5.62	6.31
05/21	-22.58	-12.74	4.69	7.7

Note: Eddy amplitude is defined as the difference between the extremum in the eddy interior and the value along the eddy periphery. The amplitude of the cyclonic (anticyclonic) eddy is negative (positive).

**Fig 4:**
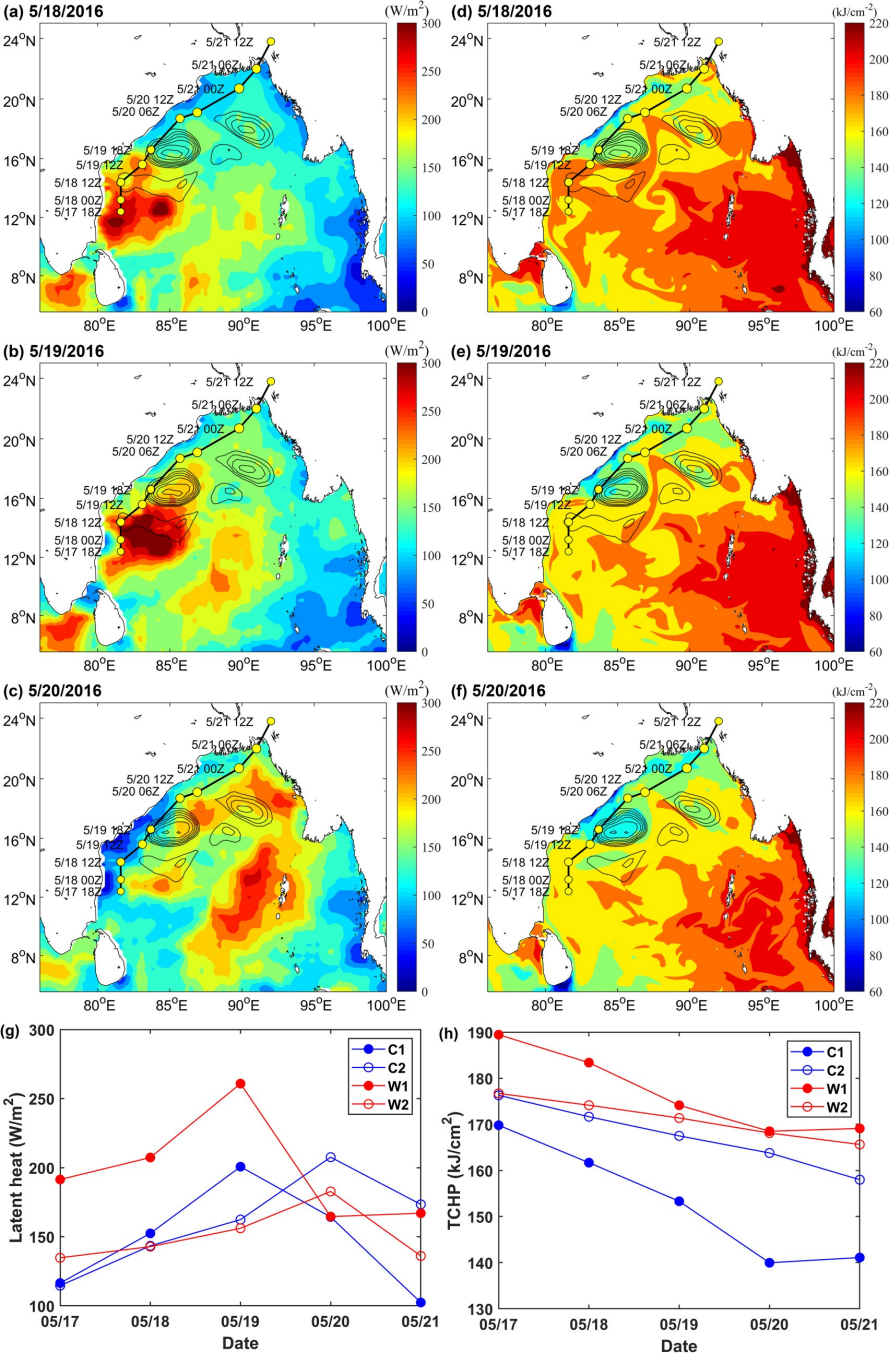
The heat changes of ocean eddies during the development of the TS. (A)-(C) show changes in latent heat flux on May 18–20, 2016, respectively; (D)-(F) show changes in TCHP on May 18–20, 2016, respectively; and (G) and (H) present line charts of the spatial average latent heat flux and TCHP of C1, C2, W1, and W2 during the development of the TS, respectively. Average values were calculated from the outermost closed contour lines of the eddies.

During the TS development, obvious thermal changes occurred in the upper ocean and especially in the ocean eddies. Overall, latent heat flux over the warm eddies was much greater than the cold eddies (Fig 4A–4C and 4G). On May 19, the maximum average latent heat flux over W1 was 260.85 W/m^2^ and that over C2 was only 200.71 W/m^2^ (Fig 4G). W2 released slightly less heat because its eddy was smaller. While latent heat flux over C2 on May 20 was considerable, it was not positioned on the track of the TS and was blocked by the W2 extension area, which had a lesser effect on the TS. After the TS passed, latent heat flux decreased significantly. Fig 4D–4F and 4H also clearly illustrate this phenomenon, in early stages of the TS, the higher TCHP of the BoB, heat levels in the ocean were larger, and the heat content of warm eddies was significantly higher than that of cold eddies. The passage of the TS caused significant heat loss in the upper ocean [43, 44]. C1 decreased from 169.8 kJ/cm^2^ on May 17 to 139.98 kJ/cm^2^ on May 20, representing a loss of 29.82 kJ/cm^2^. W1, W2, C2 also lost 20.95 kJ/cm^2^, 11.07 kJ/cm2, 9.31 kJ/cm2, respectively (Fig 4H). After Roanu passed, the ocean eddies lost their energy, and their accumulated energy had been transferred to the TS [45]. The heat of warm eddies greatly promoted the intensification of the TS, while the existence of cold eddies limited the further development of TS. At the same time, the TS also spurred dramatic sea surface cooling, and the TCHP of C1 dropped considerably. Thus, in the ocean eddies along the Roanu track, W1 first promoted the development of Roanu (W1 weakened); when the TS passed through C1, its speed was relatively fast, and the intensity was relatively weak. C1 cannot provide sufficient energy to Roanu, and then C1 suppressed and weakened the TS (C1 weakened); when Roanu passed through the W2 extension area, it was affected by continuously strengthening warm eddy W2, and TS strengthened again; C2 was farther from TS track and had little effect on TS.

## Discussion

### Mixing of ocean eddies

**Fig 5:**
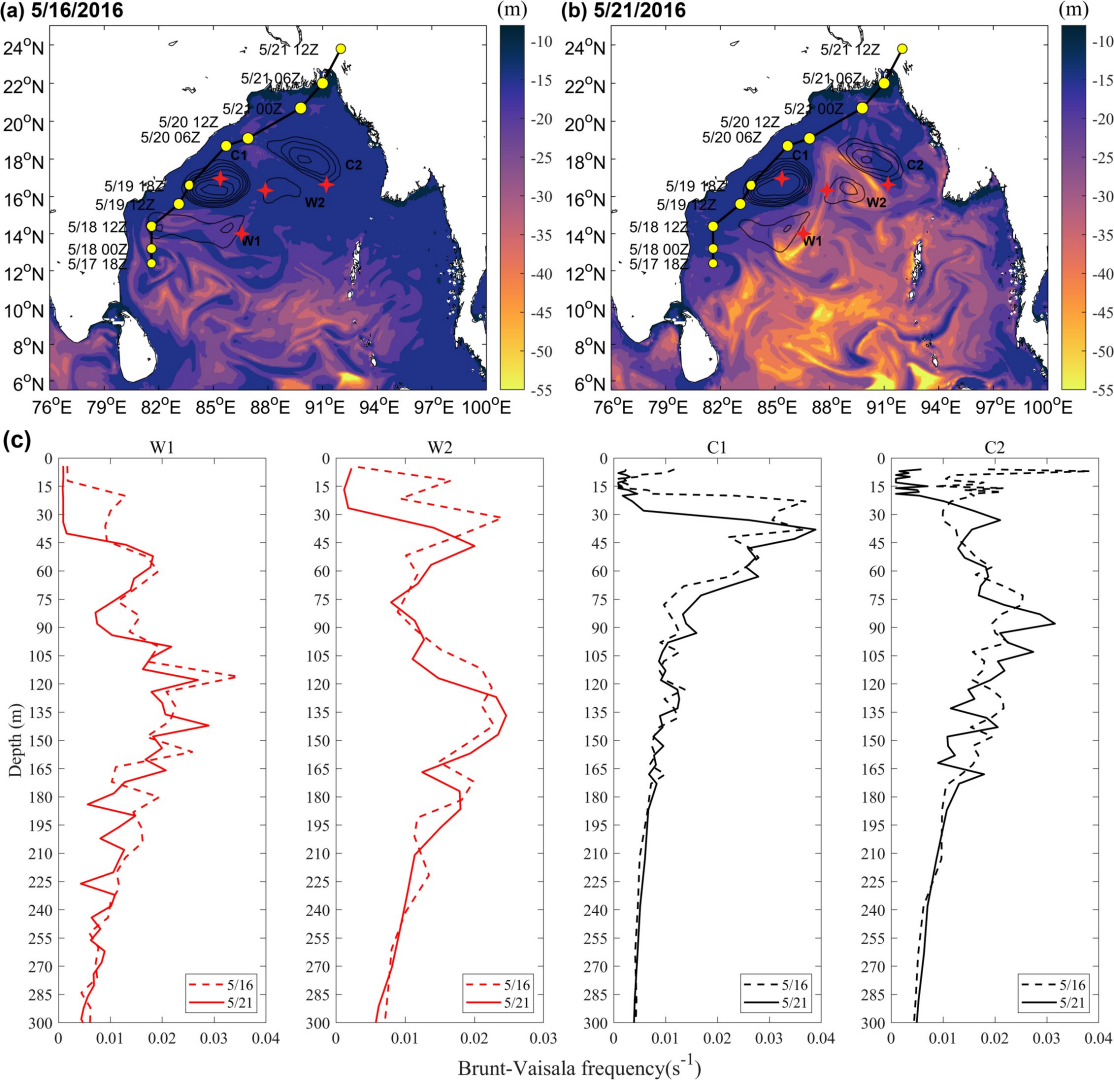
MLD and Brunt-Vaisala frequency changes before and after the TS.

Before and after the passage of the TS, the mixed layer of ocean eddies in the study area changed significantly (Fig 5). Before the passage of the TS on May 16, 2016, the mixed layer of the warm eddies (W1:16.48 m, W2:14.04 m) was deeper than that of the cold eddies (C1: 10.53 m, C2: 10.83 m) at each Argo float position. After the passage of Roanu, the mixed layer of the warm eddies deepened more obviously. On May 21, the mixed layers of W1 and W2 deepened considerably to 42.57 m and 25.33 m respectively, while the MLDs of C1 and C2 deepened slightly to only 11.14 m and 13.89 m respectively (Fig 5A and 5B).

The variation in ocean stratification shown in Fig 5C may also reflect the deepening of the mixed layer. The Brunt-Vaisala frequency of ocean eddies decreased slightly after Roanu, meaning that the TS promoted the vertical mixing of seawater and deepened mixed layer. As the BOB upper ocean was stratified significantly under the influence of rivers and precipitation, the low-salinity seawater in the surface layer was located above the deep warm seawater [46, 47], TS edge strongly negative wind stress curl anomaly (Fig 3D–3F), leading to the upper seawater convergent sinking, mixed layer thickness increased, intensifying northeastern sections of warm eddies W1 and W2 and slightly deepening the mixed layers of cold eddies C1 and C2. In addition, because the mixed layers of the warm eddies were very deep [22, 23], cooling water in the thermocline could not entrain into the mixed layer, decreasing sea surface cooling due to the TS and providing more latent heat flux for the TS (Fig 4A–4C).

**Fig 6:**
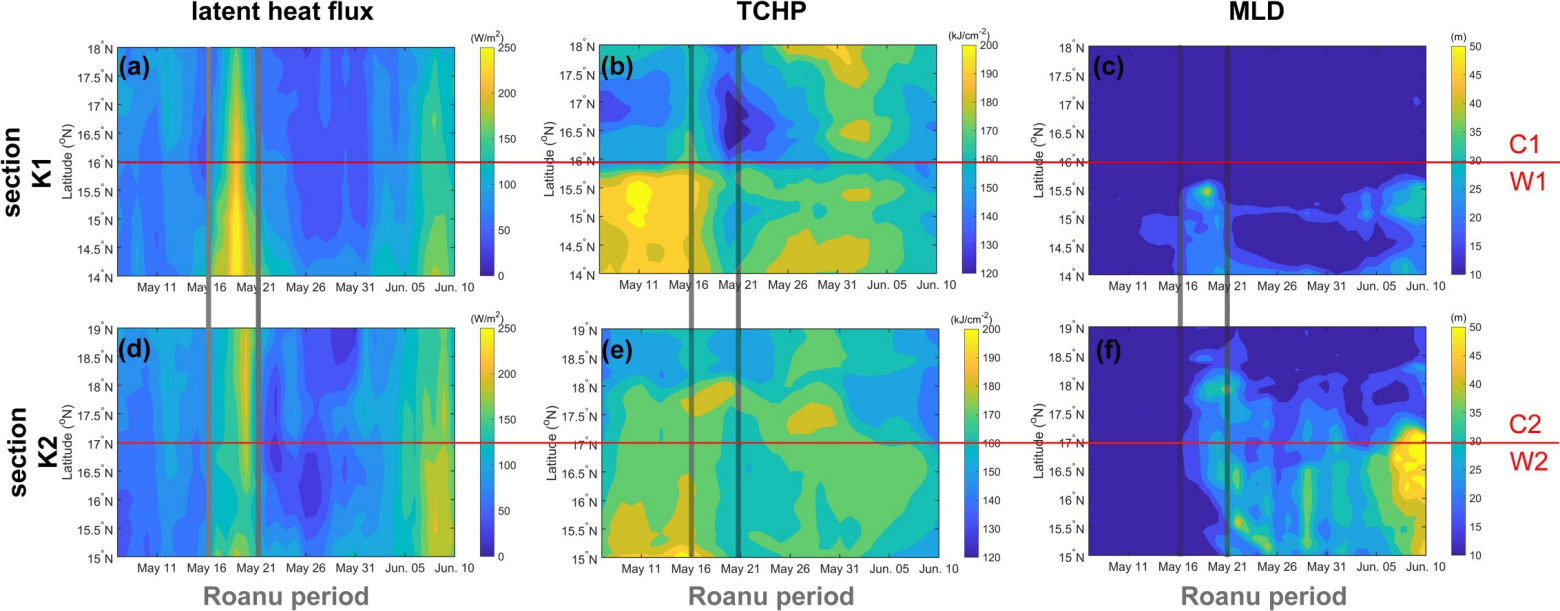
Changes of latent heat flux, TCHP and MLD on sections K1 and K2 before and after the TS (May 7 to Jun. 10, 2016). The red lines distinguish the cold eddies from the warm eddies, and two gray lines indicate the passage period of the TS.

The changes of latent heat flux, TCHP and MLD in sections K1 and K2 one month before and after the Roanu also supported the above phenomenon (Fig 6). Both sections show that the latent heat flux during Roanu was larger than that during non- Roanu, TCHP was smaller than that during non-Roanu, and MLD began to deepen continuously from May 16. Mandal et al. and Roy Chowdhury et al. [47, 48] also mentioned that heat loss and wind speed increase would cause strong air-sea flux near the ocean surface. Because C2 and W2 on section K2 were small, weak and far from the TS track, the phenomenon reflected by section K2 was not as obvious as that in section K1, but the continuous strengthening process of W2 can be clearly seen (Fig 6E and 6F). The southern part K1 was warm eddy W1, whose latent heat flux, TCHP and MLD were larger than that of C1 (Fig 6A–6C). The existence of the ocean eddies provided a favorable environment for the weakening and re-strengthening of the TS. The deeper mixed layer in W1 and W2 restricted the TS to bring a large amount of cooler water into the ocean mixed layer through vertical mixing [24], and latent heat transfer caused by warm eddies promoted TS to enhanced strongly [45], while the colder seawater in C2 reduced the latent heat flux from ocean to atmosphere [14, 49], weakening TS. Therefore, W1 enhanced the TS, C1 weakened the TS and W2 suppressed TS attenuation and re-enhanced Roanu.

### Atmospheric potential vorticity

**Fig 7:**
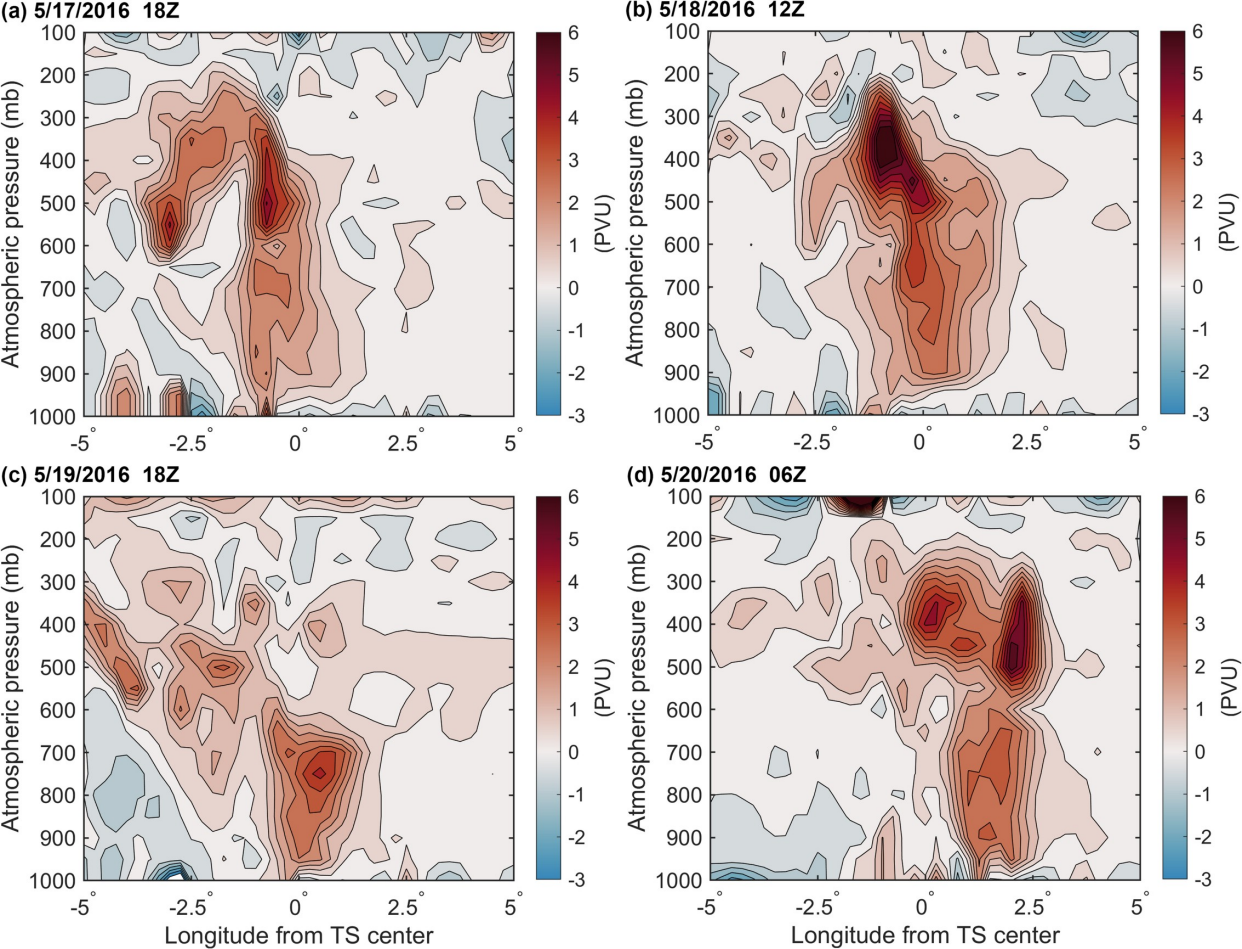
The change in the zonal vertical cross-section of the PV of ± 5 longitude over the TS center during TS development (unit: PVU, 1PVU = 10^6^ K m^2^ kg^-1^ s^-1^).

Potential vorticity is a comprehensive physical quantity based on both thermodynamic and dynamic factors that can effectively reflect the internal structure and intensity of a TS [50, 51]. When a cyclonic rotating air column contracts (converges), the air column is elongated, PV increases, rotation is accelerated, and cyclone intensity is enhanced. By contrast, when the air column expands (diverges), the air column is shortened, PV decreases, rotation slows, and cyclone intensity decreases. Fig 7 shows changes in the zonal vertical cross-section of the PV of ± 5 longitude over the TS center during TS development. At 18Z on May 17, 2016, there were high PV centers at 500 mb and 700 mb height, respectively (Fig 7A), and At 12Z on May 18, the high PV centers at 400 mb and 700 mb height, respectively, the PV at 400 mb was stronger (Fig 7B). These high PV areas were obviously independent of the upper stratospheric air and were likely the product of latent heat released over the tropical ocean (Figs 7A and 6B). When the TS passed W1 (At 12Z on May 18), a large amount of latent heat was released from the warm eddy, the PV at 400 mb could reach 7.02 PVU, and that at 700 mb could also reach 3.98 PVU. At this time, positive vorticity column elongated and vorticity increased, and the high PV in the middle layer propagated downward, leading to the deeper development of the low-layer TS (Fig 7B). At 18Z on May 19, TS passed the C1 cold eddy, the supply of latent heat was significantly reduced, the high PV center at 400 mb dissipated, the positive vorticity column became wider and shorter, and the TS weakened, leaving only a high PV center of 4.19 PVU at 750 mb (Fig 7C). At 06Z on May 20, the TS passed the extension area of W2 and was supplied with latent heat over the warm eddy; a high PV center then appeared again at 450 mb. At this time, the positive vorticity column extended and connected to the positive PV area of the tropopause, and the upper positive PV was superimposed over TS, facilitating the downward transmission of upper cold air, promoting the storage and release of potential unstable energy, intensifying the TS (Fig 7D). These PV dynamics were consistent with Bell and Montgomery’s results [52].

## Conclusions

In this paper, based on air-sea conditions, a phenomenon in which the tropical storm Roanu suddenly weakened and then recovered and intensified was analyzed using multisource reanalysis and in situ float data. In a favorable atmospheric environment where the upper layer diverged and the lower layer converged, ocean eddies had a major influence on TS development. The warm eddies W1 and W2 enhanced Roanu intensity, while the cold eddy C1 weakened Roanu intensity. C2 had a weak influence on the TS because it was not on the translation track of the TS and was blocked by the W2 extension area.

1. Warm eddy W1 supports the initial development of Roanu. At 12Z on May 18, the TS passed through W1, and positive vorticity column was elongated with high PV centers located in the middle and lower atmosphere layers. The extremum of PV was 7.02 PVU at 400 mb. The maximum average latent heat flux over warm eddy W1 reaching 260.85 W/m^2^ on May 19, 2016. After the TS, W1 lost energy, and TCHP of W1 decreased by 20.95 kJ/cm^2^.

2. Cold eddy C1 then suppressed and weakened Roanu. At 18Z on May 19, when the TS passed through the C1 cold eddy, the positive vorticity column clearly became wider and shorter, the high PV center in the middle atmosphere dissipated, and the extremum of PV in the 750 mb lower layer was only 4.19 PVU. On May 19, the supply of latent heat was obviously insufficient, and the maximum average latent heat flux over cold eddy C1 was only 200.71 W/m^2^, leading to the observed decrease in TS intensity. After Roanu, the TCHP of C1 decreased by 29.82 kJ/cm^2^.

3. Warm eddy W2 provided support for the re-enhancement of Roanu. At 06Z on May 20, 2016, the TS passed through the extension area of W2, and positive vorticity column was elongated again. The column extended to the tropopause, and PV energy was supported by both the upper atmosphere and by latent heat over W2. After the passage of the TS, the TCHP of W2 decreased by 11.07 kJ/cm^2^. W2 was supplied with energy from W1 and its amplitude increased from 4.05 cm to 7.7 cm.

4. The passage of the TS deepened the MLDs of ocean eddies, and warm eddies deepened the most. The MLDs of W1 and W2 deepened to 42.57 m and 25.33 m, respectively, and the MLDs of C1 and C2 also slightly deepened to 11.14 m and 13.89 m, respectively. The deepening of MLDs may have reduced the entrainment of cold water from thermoclines into surface sea water, reduced the cooling range of SST, and supplied more heat to the atmosphere to promote the development of the TS.

## Supporting information

S1 Data (ZIP)
